# Artificial intelligence and consistency in patient care: a large-scale longitudinal study of mammographic density assessment

**DOI:** 10.1093/bjrai/ubaf004

**Published:** 2025-03-03

**Authors:** Susan O Holley, Daniel Cardoza, Thomas P Matthews, Elisha E Tibatemwa, Rodrigo Morales Hoil, Adetunji T Toriola, Aimilia Gastounioti

**Affiliations:** Onsite Women’s Health, Nashville, TN 37203, United States; Whiterabbit.ai, Redwood City, CA 94065, United States; Whiterabbit.ai, Redwood City, CA 94065, United States; Mallinckrodt Institute of Radiology, Washington University School of Medicine, St. Louis, MO 63110, United States; Whiterabbit.ai, Redwood City, CA 94065, United States; Division of Public Health Sciences, Department of Surgery, Washington University School of Medicine, St. Louis, MO 63110, United States; Siteman Cancer Center, Barnes-Jewish Hospital, Washington University School of Medicine, St. Louis, MO 63110, United States; Mallinckrodt Institute of Radiology, Washington University School of Medicine, St. Louis, MO 63110, United States; Siteman Cancer Center, Barnes-Jewish Hospital, Washington University School of Medicine, St. Louis, MO 63110, United States

**Keywords:** breast density, artificial intelligence, mammography, breast cancer screening, longitudinal analysis

## Abstract

**Objectives:**

To assess whether use of an artificial intelligence (AI) model for mammography could result in more longitudinally consistent breast density assessments compared with interpreting radiologists.

**Methods:**

The AI model was evaluated retrospectively on a large mammography dataset including 50 sites across the United States from an outpatient radiology practice. Examinations were acquired on Hologic imaging systems between 2016 and 2021 and were interpreted by 39 radiologists (36% fellowship trained; years of experience: 2-37 years). Longitudinal patterns in 4-category breast density and binary breast density (non-dense vs. dense) were characterized for all women with at least 3 examinations (61 177 women; 214 158 examinations) as constant, descending, ascending, or bi-directional. Differences in longitudinal density patterns were assessed using paired proportion hypothesis testing.

**Results:**

The AI model produced more constant (*P* < .001) and fewer bi-directional (*P* < .001) longitudinal density patterns compared to radiologists (AI: constant 81.0%, bi-directional 4.9%; radiologists: constant 56.8%, bi-directional 15.3%). The AI density model also produced more constant (*P* < .001) and fewer bi-directional (*P* < .001) longitudinal patterns for binary breast density. These findings held in various subset analyses, which minimize (1) change in breast density (post-menopausal women, women with stable image-based BMI), (2) inter-observer variability (same radiologist), and (3) variability by radiologist’s training level (fellowship-trained radiologists).

**Conclusions:**

AI produces more longitudinally consistent breast density assessments compared with interpreting radiologists.

**Advances in knowledge:**

Our results extend the advantages of AI in breast density evaluation beyond automation and reproducibility, showing a potential path to improved longitudinal consistency and more consistent downstream care for screened women.

## Introduction

Breast density refers to the amount of fibroglandular (“dense”) tissue within the breast. Breast density tends to gradually decrease with age, parity, and tamoxifen use, while increases in breast density tend to occur when body mass index (BMI) decreases, during pregnancy, or with hormonal replacement therapy.^1–3^ Dense breast tissue can mask underlying cancers, making mammography less sensitive.^4^ Mammographic breast density is also an established independent risk factor for breast cancer, with women with dense breasts being at higher risk for breast cancer than women at otherwise similar risk levels who have less dense breasts.^5^

The most commonly used breast density assessment method in the clinical setting is visual grading, wherein breast density is classified by interpreting radiologists based on the American College of Radiology (ACR) Breast Imaging Reporting and Data System (BI-RADS^Ⓡ^).^6^ The BI-RADS^Ⓡ^ density classification includes 4 categories of breast density (a, almost entirely fatty; b, scattered areas of fibroglandular density; c, heterogeneously dense, which may obscure small masses; and d, extremely dense, which lowers the sensitivity of mammography). Higher breast density categories have been consistently associated with increasing levels of breast cancer risk.^7^^,^^8^ Moreover, inclusion of breast density in clinical risk prediction models, such as the Tyrer-Cuzick model, has been shown to improve predictive accuracy.^9^ However, BI-RADS^Ⓡ^ density classification is a subjective process with substantial inter- or intra-radiologist discrepancies^10^ that may have critical implications for a woman’s downstream care. In particular, inconsistent density assessments over time can lead to women receiving contrary recommendations for supplemental imaging with ultrasound or MRI. Furthermore, discrepancies in density assessments over time may interfere with the identification of longitudinal density trajectories associated with increased levels of breast cancer risk.^11–13^

To enhance reproducibility and robustness in breast density assessment, various artificial intelligence (AI) models have been proposed to automatically classify mammographic images into BI-RADS^Ⓡ^ density categories.^14–19^ Despite their promising performances,^20^ so far breast density AI models have been assessed primarily in studies using a single examination for each woman. Therefore, their potential to enhance consistency in breast density assessment over time remains largely unexplored.

This study aims to address this question by using a large, multi-site mammographic screening cohort to assess whether use of a commercially available breast density AI model^17^ (WRDensity v1.1; Whiterabbit.ai, Santa Clara, CA) could result in more longitudinally consistent BI-RADS^Ⓡ^ breast density assessments for women compared with interpreting radiologists.

## Methods

### Study dataset

**Figure 1.**
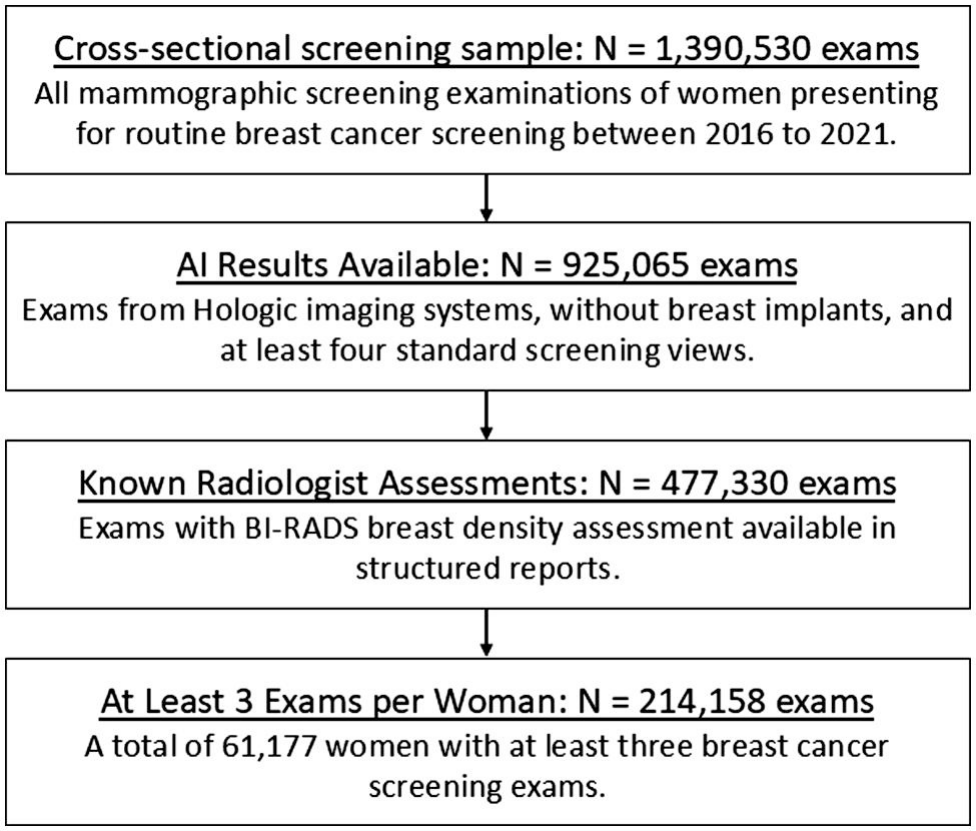
Flowchart shows inclusion and exclusion criteria for the cross-sectional screening sample analysed in our study.

In this institutional review board-approved, Health Insurance Portability and Protection Act-compliant study under a waiver of consent, we retrospectively analysed a cross-sectional cohort of women who underwent at least 3 breast cancer screening examinations between January 1, 2016 and June 11, 2021 at an outpatient radiology practice (Onsite Women’s Health), using 50 sites across the United States (Figure 1). All women with breast implants were excluded. Examinations that were not acquired on Hologic imaging systems (Selenia Dimensions; Hologic, Inc.) or that did not have at least 1 mediolateral oblique (MLO) and 1 craniocaudal (CC) view per breast were also excluded. All screening examinations were performed with a combination of digital breast tomosynthesis (DBT) with digital mammography (DM) or 2D synthetic mammography (SM) images.

Images from standard acquisition angles (CC and MLO) obtained at mammographic screening examinations were used in our analysis. Age at screening was available for all examinations in electronic medical records. BMI at the time of each screening examination was also retrieved from the electronic medical record; when unavailable, BMI was approximated using image-based measures of breast fat and thickness (see Supplemental Materials).

### Breast density assessment

The screening examinations in our dataset were interpreted by 1 of 39 board-certified radiologists who specialized in breast imaging and who had between 2 and 37 years of experience (36% with fellowship training). BI-RADS^Ⓡ^ density categories, assigned to each screening examination by the interpreting radiologist, were extracted from a structured mammography information system. The screening examinations were re-analysed for breast density using an AI model that provides an ACR BI-RADS^Ⓡ^ breast density category from DM or SM images. The AI density model was previously developed using deep learning based on over 600 000 DM and SM images from a large academic breast cancer screening practice.^17^ Screening examinations with indications of failed processing by the AI density model were excluded (Figure 1).

### Statistical analysis

Longitudinal breast density patterns were characterized for all women as either constant, descending, ascending, or bi-directional, using 4-category breast density (BI-RADS^Ⓡ^ categories a-d) and binary breast density assessments (non-dense: BI-RADS^Ⓡ^ a and b; dense: BI-RADS^Ⓡ^ c and d). Given the short time window of our dataset (5 years), constant longitudinal patterns in breast density are the most physiologically likely to occur, while bi-directional longitudinal patterns in breast density are likely due to intra- and inter-reader variability for radiologists or limited robustness of the AI model. Also, Boyd et al.^21^ previously showed small decreases in area percent breast density over a 5-year period, especially for women not experiencing menopause. Therefore, our study focused primarily on constant and bi-directional longitudinal breast density patterns, and differences between the AI density model and interpreting radiologists were assessed using McNemar paired proportion hypothesis testing.^22^ In the main analysis, we assessed differences in the overall dataset, and in sub-analyses, we assessed differences in 4 overlapping subgroups of our dataset consisting of (1) post-menopausal women (women older than 55 years at first examination in the dataset), (2) women with stable image-based BMI (ie, proportional change of BMI <5%^23^) between examinations (see Supplemental Materials), (3) women with examinations interpreted by the same radiologist, and (4) women with examinations interpreted by fellowship-trained radiologists. All statistical analyses were performed by using software (Python 3.8, python.org; Stata 17; StataCorp, College Station, TX, United States), and a *P*-value of .05 or less indicated statistical significance. Because of the large sample size, we had substantial power to detect differences in longitudinal breast density patterns in the main and subgroup analyses.

## Results

Our study dataset was composed of 214 158 screening examinations (92.9% DM; 7.1% SM) from 61 177 women (mean age, 55.6 years; standard deviation, 10.3 years) (Table 1). A total of 37 156 women had 3 screening examinations (60.7% of our dataset), 17 618 women had 4 screening examinations (28.8%), and 6403 women had 5 or 6 screening examinations (10.5%). The overall BI-RADS^Ⓡ^ breast density distributions across all examinations exhibit small but detectable differences (*P* < .001) for the AI density model (a: 9.5%, b: 48.7%, c: 36.1%, d: 5.7%) and the interpreting radiologists (a: 9.0%, b: 45.1%, c: 38.1%, d: 7.7%).

**Table 1.** Study dataset characteristics.

	AI density model	Interpreting radiologists	
Age (years) at screening^a^	55.6 (10.3)		
BMI (kg/m^2^) at screening^a^^,^^b^	28.3 (4.9)		
Menopausal status			
Postmenopausal (age >55 years)	95 509 (44.6%)		
Pre/peri-menopausal (age ≤55 years)	118 649 (55.45%)		
BI-RADS^Ⓡ^ density			<0.001
a	20 407 (9.5%)	19 295 (9.0%)	
b	104 279 (48.7%)	96 650 (45.1%)	
c	77 324 (36.1%)	81 695 (38.1%)	
d	12 148 (5.7%)	16 518 (7.7%)	

Data in parentheses are percentages.

a Mean (SD).

b BMI was approximated using image-based measures of breast fat and thickness (see Supplemental Materials).

Abbreviations: AI = artificial intelligence; BMI = body mass index; BI-RADS = Breast Imaging Reporting and Data System.

**Figure 2.**
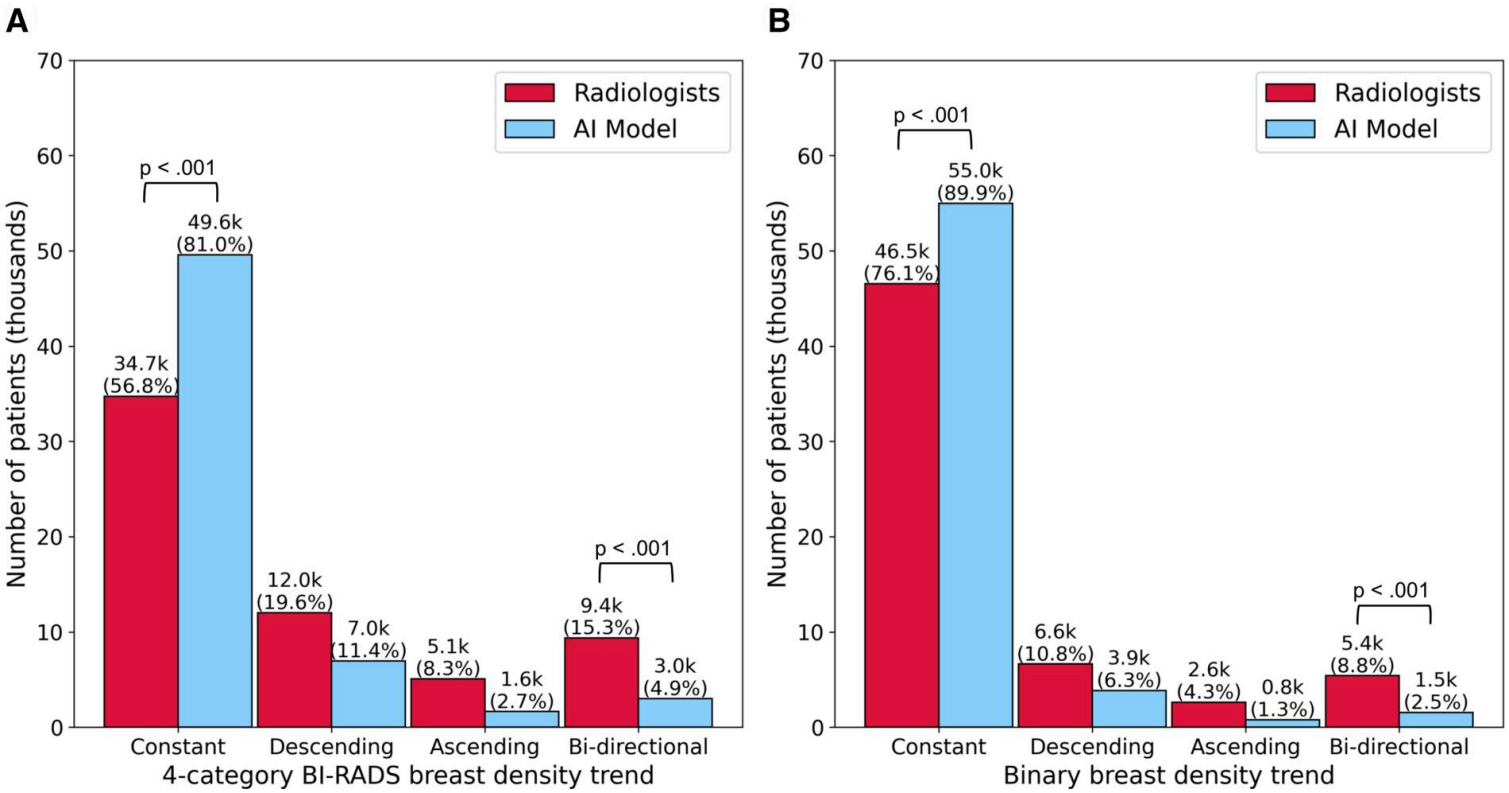
Longitudinal patterns in (A) 4-category breast density and (B) binary breast density (dense vs. non-dense).

**Figure 3.**
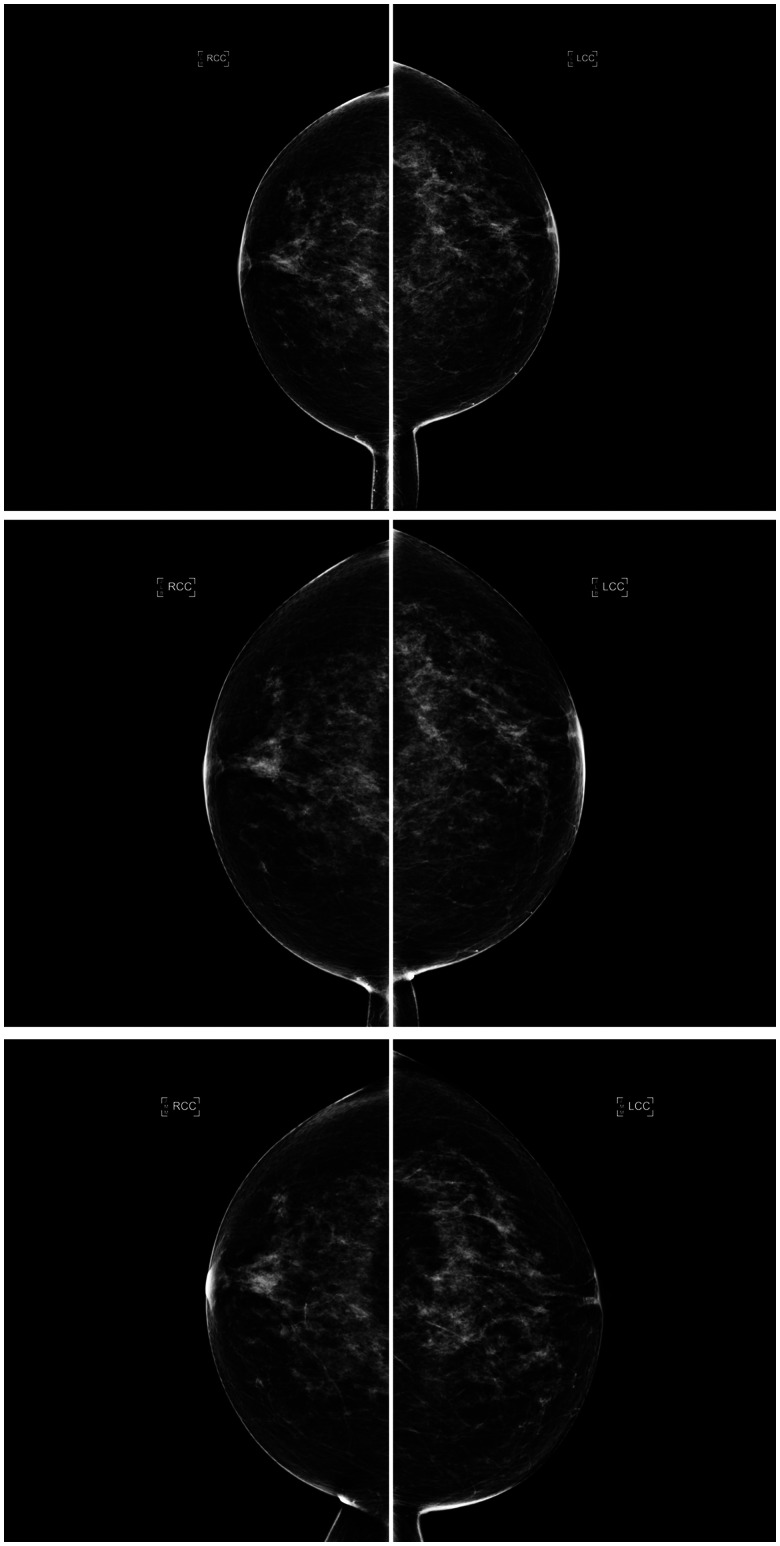
Consecutive craniocaudal mammographic views acquired in the year 2019 (top), 1 year later (middle), and 2 years later (bottom) of the same woman. An ascending longitudinal density pattern was produced by the interpreting radiologists due to inter/intra-observer variability who assigned BI-RADS density categories b, c, and d, respectively, whereas the AI density model assigned BI-RADS density category c to all 3 examinations, producing a constant longitudinal density pattern. Abbreviations: BI-RADS = Breast Imaging Reporting and Data System; AI = artificial intelligence.

**Figure 4.**
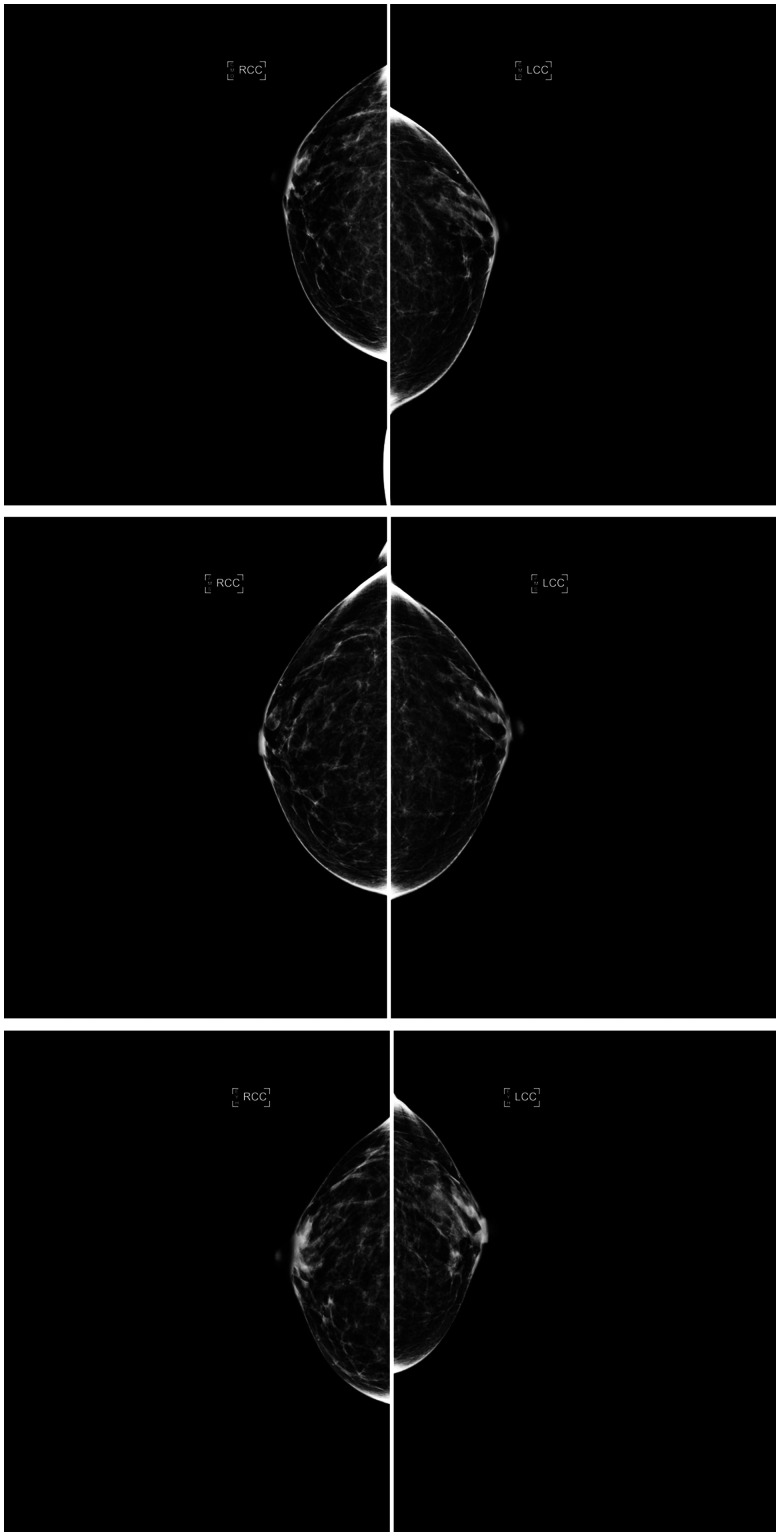
Consecutive craniocaudal mammographic views acquired in the year 2017 (top), 3 years later (middle), and 4 years later (bottom) of the same woman. The interpreting radiologists and the AI density model agreed on the same ascending longitudinal density pattern and assigned BI-RADS density categories b, b, and c, respectively, capturing potential weight loss between the last 2 years (as suggested by the reduction in compressed breast thickness of the left and right breasts from 4.9/5.0 cm to 3.1/3.1 cm). Abbreviations: AI = artificial intelligence; BI-RADS = Breast Imaging Reporting and Data System.

**Figure 5.**
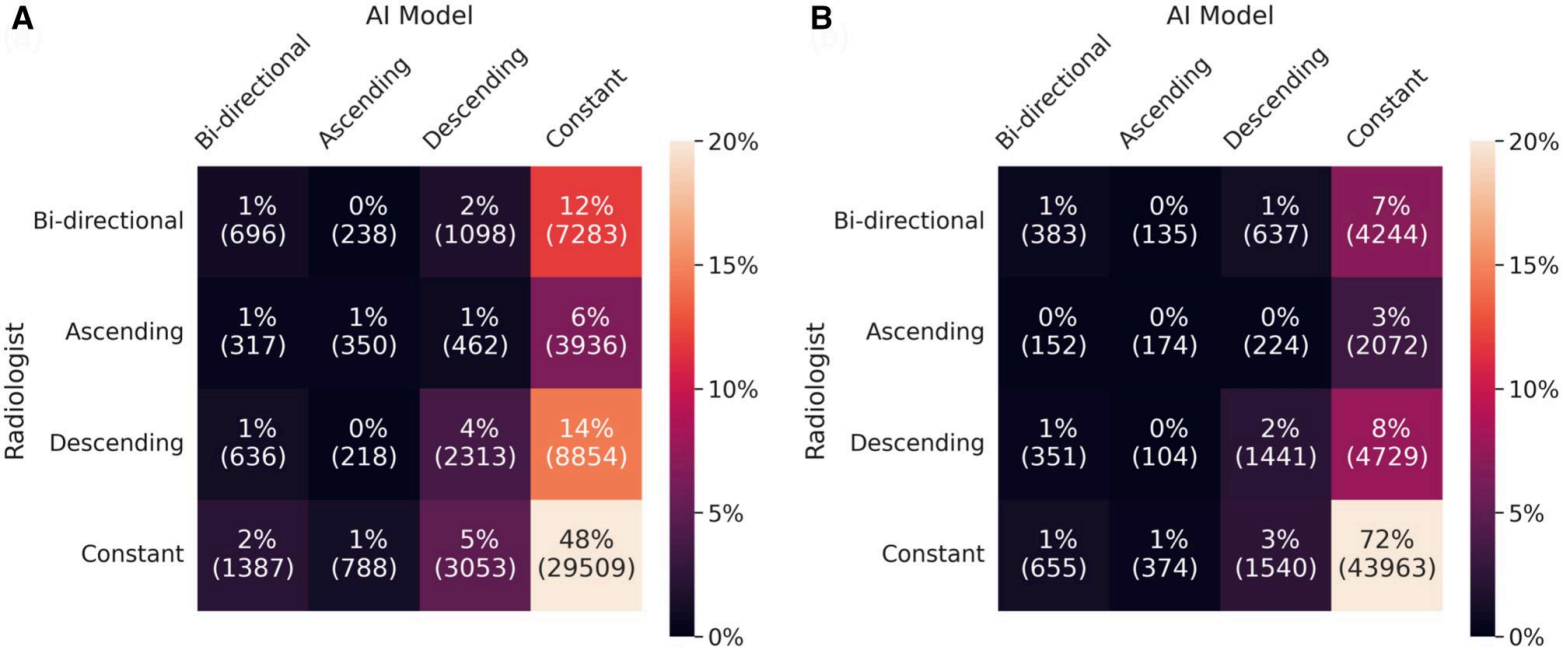
Confusion matrices for longitudinal patterns in (A) 4-category breast density and (B) binary breast density (dense vs. non-dense).

**Figure 6.**
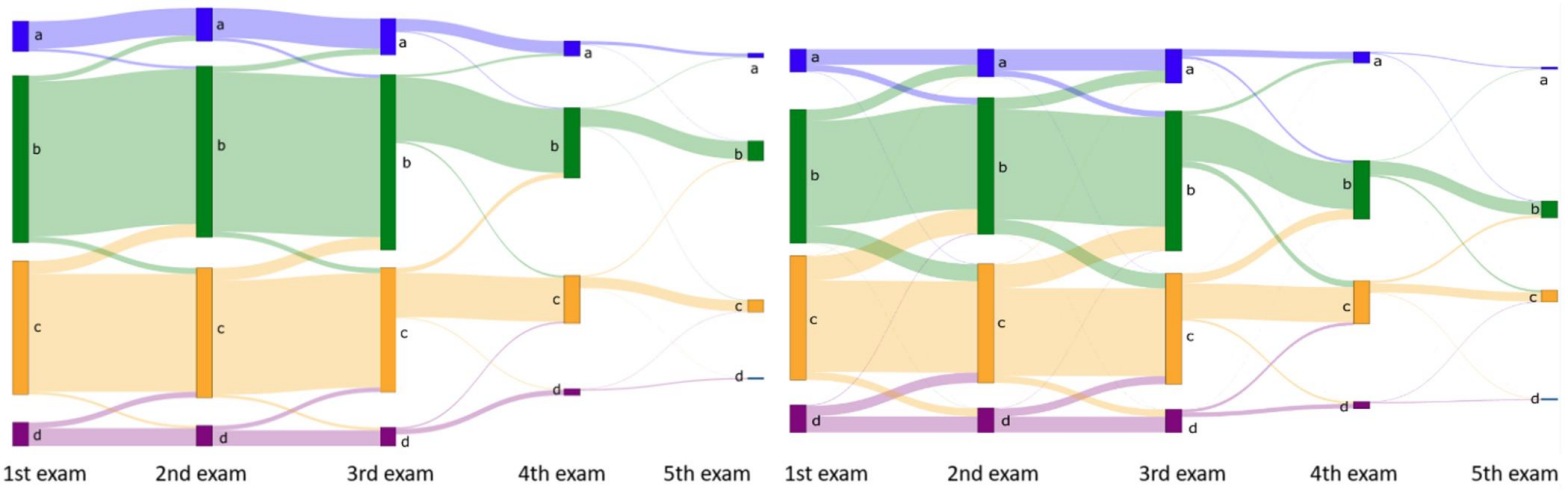
Sankey plots showing changes in 4-category breast density from women’s first to last screening examination, based on (left) the AI density model and (right) interpreting radiologists. Abbreviation: AI = artificial intelligence.

The AI density model produced 10.4% fewer bi-directional (*P* < .001) and 24.2% more constant (*P* < .001) longitudinal patterns in 4-category breast density compared to interpreting radiologists (Figure 2A). The AI density model also produced 6.3% fewer bi-directional (*P* < .001) and 13.8% more constant (*P* < .001) longitudinal patterns for binary breast density (Figure 2B). Examples of longitudinal breast density patterns detected by the radiologists and the AI density model are shown in Figures 3 and 4. For many women, the interpreting radiologists and the AI model agreed upon a constant longitudinal pattern of breast density in both 4-category and binary density contexts (Figure 5); however, most disagreements occurred when the AI model identified a constant longitudinal density pattern and interpreting radiologists did not (Figure 5). Detailed changes in the proportions of the 4 breast density categories and in the proportion of dense vs non-dense breasts across consecutive screenings are shown in Figure 6.

Similar conclusions held for all subgroups of our cohort, with the AI model consistently producing fewer bi-directional and more constant longitudinal density patterns compared to interpreting radiologists (Tables 2 and 3). Interestingly, when examinations were reviewed by the same radiologist, by fellowship-trained radiologists, or when focusing on women with anticipated minimal changes in their breast parenchymal patterns over the 5-year window of our study (ie, women with stable image-based BMI), the interpreting radiologists produced fewer bi-directional and more constant longitudinal density patterns relative to their performance on the full dataset, yet still demonstrated less consistency compared to the AI model for these subgroups (Tables 2 and 3).

**Table 2.** Longitudinal patterns in 4-category breast density for the AI density model and interpreting radiologists in the full cohort and in subgroups of women.

Longitudinal density pattern	AI density model	Interpreting radiologists	*P*-value^a^
Full cohort (*N* = 61 177)			
Constant	49 575 (81.0%)	34 736 (56.8%)	<.001
Ascending	1637 (2.7%)	5065 (8.3%)	
Descending	6953 (11.4%)	12 021 (19.6%)	
Bi-directional	3012 (4.9%)	9355 (15.3%)	<.001
Postmenopausal women (*N* = 27 268)			
Constant	23 246 (85.3%)	15 603 (57.2%)	<.001
Ascending	733 (2.7%)	2405 (8.8%)	
Descending	2093 (7.7%)	5018 (18.4%)	
Bi-directional	1196 (4.4%)	4242 (15.6%)	<.001
Women with stable image-based BMI (*N* = 4477)			
Constant	3817 (85.3%)	2823 (63.1%)	<.001
Ascending	104 (2.4%)	356 (8.0%)	
Descending	377 (8.4%)	887 (19.8%)	
Bi-directional	179 (5.0%)	411 (9.2%)	<.001
Women with examinations interpreted by the same radiologist (*N* = 21 216)			
Constant	17 267 (81.4%)	14 218 (67.0%)	<.001
Ascending	584 (2.8%)	1368 (6.4%)	
Descending	2404 (11.3%)	3734 (17.6%)	
Bi-directional	961 (4.5%)	1896 (8.9%)	<.001
Women with examinations interpreted by fellowship-trained radiologists (*N* = 22 462)			
Constant	19 696 (87.7%)	16 122 (71.8%)	<.001
Ascending	312 (6.4%)	1286 (5.7%)	
Descending	1446 (1.4%)	1715 (7.6%)	
Bi-directional	1008 (4.5%)	3339 (14.9%)	<.001

Data in parentheses are percentages.

a *P*-value for paired proportion hypothesis testing between the AI density model and interpreting radiologists.

Abbreviations: AI = artificial intelligence; BMI = body mass index.

**Table 3.** Longitudinal patterns in binary breast density (dense vs. non-dense) for the AI density model and interpreting radiologists in the full cohort and in subgroups of women.

Longitudinal density pattern	AI density model	Interpreting radiologists	*P*-value^a^
Full cohort (*N* = 61 177)			
Constant	54 997 (89.9%)	46 531 (76.1%)	<.001
Ascending	3853 (6.3%)	6625 (10.8%)	
Descending	785 (1.3%)	2622 (4.3%)	
Bi-directional	1542 (2.5%)	5399 (8.8%)	<.001
Postmenopausal women (*N* = 27 268)			
Constant	25 103 (92.1%)	20 902 (76.7%)	<.001
Ascending	373 (1.4%)	1278 (4.7%)	
Descending	1163 (4.3%)	2627 (9.6%)	
Bi-directional	629 (2.3%)	2461 (9.0%)	<.001
Women with stable image-based BMI (*N* = 4477)			
Constant	4132 (92.3%)	3631 (81.1%)	<.001
Ascending	45 (1.0%)	147 (3.3%)	
Descending	215 (4.8%)	457 (10.2%)	
Bi-directional	85 (1.9%)	242 (5.4%)	<.001
Women with examinations interpreted by the same radiologist (*N* = 21 216)			
Constant	19 137 (90.2%)	17 434 (82.2%)	<.001
Ascending	267 (1.3%)	706 (3.3%)	
Descending	1329 (6.3%)	1902 (9.0%)	
Bi-directional	483 (2.3%)	1174 (5.5%)	<.001
Women with examinations interpreted by fellowship-trained radiologists (*N* = 22 462)			
Constant	21 007 (93.5%)	18 096 (80.6%)	<.001
Ascending	136 (0.6%)	784 (3.5%)	
Descending	809 (3.6%)	1228 (5.5%)	
Bi-directional	510 (2.3%)	2354 (10.5%)	<.001

Data in parentheses are percentages.

a *P*-value for paired proportion hypothesis testing between the AI density model and interpreting radiologists.

Abbreviations: AI = artificial intelligence; BMI = body mass index.

## Discussion

The potential of AI to enhance consistency in clinical breast density assessment over time is largely unexplored. Our study addressed this question by assessing whether use of a previously validated AI model^17^ could result in more longitudinally consistent BI-RADS^Ⓡ^ breast density assessments for women compared with interpreting radiologists. Our data from a large, multi-site screening cohort of over 61 000 women, each with 3-6 mammographic screening examinations acquired over a 5-year period, showed that the AI model provides more longitudinally consistent breast density assessments compared to radiologists. This was seen with both 4-category breast density and binary breast density (dense vs non-dense) assessments, where, compared to radiologists, the AI model produced significantly more constant breast density patterns over time (4-category breast density: 81.0% vs 56.8%, *P* < .001; binary breast density: 89.9% vs 76.1%, *P* < .001) and significantly fewer bi-directional breast density patterns over time (4-category breast density: 4.9% vs 15.3%, *P* < .001; binary breast density: 2.5% vs 8.8%, *P* < .001). These same findings held in various sub-analyses focusing on examinations reviewed by the same radiologist/fellowship-trained radiologists or focusing on women with anticipated minimal changes in their breast parenchymal patterns over the 5-year window of our study (ie, post-menopausal women and women with stable image-based BMI). With a national breast density law in place^24^ and breast density playing a key role in breast cancer risk assessment and supplemental screening recommendations,^25^ having more consistent breast density assessment over time could lead to more consistent downstream care, in particular for dense-breasted and high-risk women.

Besides its substantial clinical impact, our study could also benefit future research on longitudinal breast density changes and their associations with breast cancer risk. Previous studies^11^^,^^13^^,^^26^^,^^27^ have consistently shown that longitudinal changes in BI-RADS^Ⓡ^ breast density are more strongly associated with breast cancer risk than a single measure of BI-RADS^Ⓡ^ breast density, as well as the fact that a reduction in BI-RADS^Ⓡ^ breast density to a lower category reduces breast cancer risk relative to a density that remains stable or increases. However, the limited ability of these studies to decouple actual longitudinal density changes from longitudinal density changes due to inter- or intra-radiologist discrepancies^10^^,^^28^ is a major drawback. This drawback could be addressed with more longitudinally consistent BI-RADS^Ⓡ^ breast density assessments provided by AI models, shedding more light into the identification of longitudinal density trajectories associated with increased levels of breast cancer risk.

A strength of our study was access to a large dataset, with a wide variety of clinics and interpreting radiologists, while being homogeneous in vendor and imaging system (Selenia Dimensions; Hologic, Inc.). Another major strength of our study was the use of a well-validated breast density AI model that supports both DM and the newer SM format acquired with DBT, which allows generalizability of our results to the current standard of breast cancer screening in the United States.^29^ Finally, in assessing consistency in longitudinal breast density assessment in both 4-category and binary breast density (dense vs non-dense) contexts, our study offers preliminary evidence about potential critical implications of inconsistent density assessments over time for a woman’s downstream care.

Certain limitations should also be noted. We did not have access to more extensive demographic data, such as self-reported race/ethnicity. Therefore, although estimation from clinic zip codes suggests that our study cohort was racially diverse (White: 71%; Black: 21%; Asian: 4%; Other: 4%), we could not stratify our analyses by race. Moreover, we did not have universal access to BMI collected during attendance at screening or individual breast cancer risk assessments. To partially address this limitation, we approximated BMI using image-based breast fat and thickness data, previously shown to provide a suitable alternative to clinically acquired weight and BMI.^30–32^ Last, compared to DM, the SM portion of our dataset was much smaller. In future studies, we aim to validate our findings in larger screening cohorts with SM. Future evaluations will also involve stratifications by race, as well as inclusion of BMI and other established breast cancer risk factors towards studying potential implications of differences in longitudinal breast density patterns on women’s risk trajectories.

Maintaining consistent AI performance over diverse patient populations, varying imaging settings, and long time horizons is a major challenge across several imaging AI applications, including AI models for mammography. For instance, previous studies have shown substantial effects of patient characteristics on AI performance in breast cancer detection with DM and DBT,^33–35^ variable performances of different AI software for screening mammography,^36^ as well as AI temporal quality degradation in dynamic clinical settings that involve multiple vendors, imaging systems, and AI models.^37^ Therefore, our future work will focus on assessing the durability of our findings over diverse patient populations, multiple mammographic imaging vendors/systems, and advancements in AI models that provide BI-RADS or continuous breast density metrics. Future prospective studies on clinically used breast density AI models could also elucidate whether interpreting radiologists would more consistently assess breast density over time with assistance of AI, which was not tested in our study.

In conclusion, in a screening cohort of over 61 000 women, each with multiple mammographic screening examinations of a single vendor acquired over a 5-year period, the breast density AI model produced more longitudinally consistent BI-RADS^Ⓡ^ breast density assessments for women compared with interpreting radiologists, with fewer bi-directional and more constant assessments over time. Our results extend the advantages of AI in BI-RADS^Ⓡ^ breast density evaluation beyond automation and reproducibility,^20^ showing a potential path to improved longitudinal consistency and more consistent downstream care for screened women.

## Supplementary Material

ubaf004_Supplementary_Data

## References

[1] Azam S, Eriksson M, Sjölander A, et al Mammographic density change and risk of breast cancer. J Natl Cancer Inst. 2020;112:391-399. 31298705 10.1093/jnci/djz149 PMC7156933

[2] Burton A, Maskarinec G, Perez-Gomez B, et al Mammographic density and ageing: a collaborative pooled analysis of cross-sectional data from 22 countries worldwide. PLoS Med. 2017;14:e1002335. 28666001 10.1371/journal.pmed.1002335 PMC5493289

[3] Kelemen LE, Pankratz VS, Sellers TA, et al Age-specific trends in mammographic density: the Minnesota Breast Cancer Family Study. Am J Epidemiol. 2008;167:1027-1036. 18385204 10.1093/aje/kwn063

[4] Krishnan K, Baglietto L, Apicella C, et al Mammographic density and risk of breast cancer by mode of detection and tumor size: a case-control study. Breast Cancer Res. 2016;18:63. 27316945 10.1186/s13058-016-0722-4 PMC4912759

[5] McCormack VA, dos Santos Silva I. Breast density and parenchymal patterns as markers of breast cancer risk: a meta-analysis. Cancer Epidemiol Biomarkers Prev. 2006;15:1159-1169. 16775176 10.1158/1055-9965.EPI-06-0034

[6] D'Orsi CJ. ACR BI-RADS Atlas: Breast Imaging Reporting and Data System. American College of Radiology; 2013.

[7] Kerlikowske K, Ma L, Scott CG, et al Combining quantitative and qualitative breast density measures to assess breast cancer risk. Breast Cancer Res. 2017;19:97. 28830497 10.1186/s13058-017-0887-5 PMC5567482

[8] Kerlikowske K, Zhu W, Tosteson ANA, et al; Breast Cancer Surveillance Consortium. Identifying women with dense breasts at high risk for interval cancer: a cohort study. Ann Intern Med. 2015;162:673-681. 25984843 10.7326/M14-1465 PMC4443857

[9] Vilmun BM, Vejborg I, Lynge E, et al Impact of adding breast density to breast cancer risk models: a systematic review. Eur J Radiol. 2020;127:109019. 32361308 10.1016/j.ejrad.2020.109019

[10] Sprague BL, Conant EF, Onega T, et al; PROSPR Consortium. Variation in mammographic breast density assessments among radiologists in clinical practice: a multicenter observational study. Ann Intern Med. 2016;165:457-464. 27428568 10.7326/M15-2934 PMC5050130

[11] Kerlikowske K, Ichikawa L, Miglioretti DL, et al; National Institutes of Health Breast Cancer Surveillance Consortium. Longitudinal measurement of clinical mammographic breast density to improve estimation of breast cancer risk. J Natl Cancer Inst. 2007;99:386-395. 17341730 10.1093/jnci/djk066

[12] Kerlikowske K, Gard CC, Sprague BL, Tice JA, Miglioretti DL; Breast Cancer Surveillance Consortium. One versus two breast density measures to predict 5- and 10-year breast cancer risk. Cancer Epidemiol Biomarkers Prev. 2015;24:889-897. 25824444 10.1158/1055-9965.EPI-15-0035 PMC4452451

[13] Tran TXM, Kim S, Song H, Lee E, Park B. Association of longitudinal mammographic breast density changes with subsequent breast cancer risk. Radiology. 2023;306:e220291. 36125380 10.1148/radiol.220291

[14] Lehman CD, Yala A, Schuster T, et al Mammographic breast density assessment using deep learning: clinical implementation. Radiology. 2019;290:52-58. 30325282 10.1148/radiol.2018180694

[15] Chang K, Beers AL, Brink L, et al Multi-institutional assessment and crowdsourcing evaluation of deep learning for automated classification of breast density. J Am Coll Radiol. 2020;17:1653-1662. 32592660 10.1016/j.jacr.2020.05.015 PMC10757768

[16] Ciritsis A, Rossi C, Vittoria De Martini I, et al Determination of mammographic breast density using a deep convolutional neural network. Br J Radiol. 2019;92:20180691. 30209957 10.1259/bjr.20180691 PMC6435091

[17] Matthews TP, Singh S, Mombourquette B, et al A multi-site study of a breast density deep learning model for full-field digital mammography images and synthetic mammography images. Radiol Artif Intell. 2021;3:e200015. 33937850 10.1148/ryai.2020200015 PMC8082294

[18] Sexauer R, Hejduk P, Borkowski K, et al Diagnostic accuracy of automated ACR BI-RADS breast density classification using deep convolutional neural networks. Eur Radiol. 2023;33:4589-4596. 36856841 10.1007/s00330-023-09474-7 PMC10289992

[19] Tari DU, Santonastaso R, De Lucia DR, Santarsiere M, Pinto F. Breast density evaluation according to BI-RADS 5th edition on digital breast tomosynthesis: AI automated assessment versus human visual assessment. J Pers Med. 2023;13:609. 37108994 10.3390/jpm13040609 PMC10146726

[20] Gastounioti A, Desai S, Ahluwalia VS, Conant EF, Kontos D. Artificial intelligence in mammographic phenotyping of breast cancer risk: a narrative review. Breast Cancer Res. 2022;24:14. 35184757 10.1186/s13058-022-01509-z PMC8859891

[21] Boyd N, Martin L, Stone J, Little L, Minkin S, Yaffe M. A longitudinal study of the effects of menopause on mammographic features. Cancer Epidemiol Biomarkers Prev. 2002;11:1048-1053. 12376506

[22] Newcombe RG. Interval estimation for the difference between independent proportions: comparison of eleven methods. Stat Med. 1998;17:873-890. 9595617 10.1002/(sici)1097-0258(19980430)17:8<873::aid-sim779>3.0.co;2-i

[23] Svärd A, Lahti J, Roos E, et al Obesity, change of body mass index and subsequent physical and mental health functioning: a 12-year follow-up study among ageing employees. BMC Public Health. 2017;17:816-810. 29041966 10.1186/s12889-017-4828-0 PMC5644067

[24] U.S. Food and Drug Administration. FDA Updates Mammography Regulations to Require Reporting of Breast Density Information and Enhance Facility Oversight. 2023. Accessed January 9, 2024. https://www.fda.gov/news-events/press-announcements/fda-updates-mammography-regulations-require-reporting-breast-density-information-and-enhance

[25] Destounis SV, Santacroce A, Arieno A. Update on breast density, risk estimation, and supplemental screening. Am J Roentgenol. 2020;214:296-305. 31743049 10.2214/AJR.19.21994

[26] Kim S, Park B. Association between changes in mammographic density category and the risk of breast cancer: a nationwide cohort study in East‐Asian women. Int J Cancer. 2021;148:2674-2684. 33368233 10.1002/ijc.33455

[27] Román M, Sala M, Baré M, et al; BELE Study Group. Changes in mammographic density over time and the risk of breast cancer: an observational cohort study. Breast. 2019;46:108-115. 31132476 10.1016/j.breast.2019.04.007

[28] Spayne MC, Gard CC, Skelly J, Miglioretti DL, Vacek PM, Geller BM. Reproducibility of BI‐RADS breast density measures among community radiologists: a prospective cohort study. Breast J. 2012;18:326-333. 22607064 10.1111/j.1524-4741.2012.01250.x PMC3660069

[29] Conant EF, Talley MM, Parghi CR, et al Mammographic screening in routine practice: multisite study of digital breast tomosynthesis and digital mammography screenings. Radiology. 2023;307:e221571. 36916891 10.1148/radiol.221571

[30] Nair KP, Harkness EF, Gadde S, et al The impact of using weight estimated from mammographic images vs self-reported weight on breast cancer risk calculation. Proc SPIE Int Soc Opt Eng. 2017;10134:101342V. 10.1117/12.2255619 PMC7612113 34925706

[31] Hudson S, Vik Hjerkind K, Vinnicombe S, et al Adjusting for BMI in analyses of volumetric mammographic density and breast cancer risk. Breast Cancer Res. 2018;20:156. 30594212 10.1186/s13058-018-1078-8 PMC6311032

[32] Alimujiang A, Appleton C, Colditz GA, Toriola AT. Adiposity during early adulthood, changes in adiposity during adulthood, attained adiposity, and mammographic density among premenopausal women. Breast Cancer Res Treat. 2017;166:197-206. 28702890 10.1007/s10549-017-4384-4

[33] Nguyen DL, Ren Y, Jones TM, Thomas SM, Lo JY, Grimm LJ. Patient characteristics impact performance of AI algorithm in interpreting negative screening digital breast tomosynthesis studies. Radiology. 2024;311:e232286. 38771177 10.1148/radiol.232286 PMC11140531

[34] Homsi N, Chung M. Patient characteristics impact false positives in AI interpretation of true-negative screening breast tomosynthesis examinations. Radiol Imaging Cancer. 2024;6:e249015. 39150358 10.1148/rycan.249015 PMC11443469

[35] Hsu W, Hippe DS, Nakhaei N, et al External validation of an ensemble model for automated mammography interpretation by artificial intelligence. JAMA Network Open. 2022;5:e2242343. 36409497 10.1001/jamanetworkopen.2022.42343 PMC9679879

[36] Salim M, Wåhlin E, Dembrower K, et al External evaluation of 3 commercial artificial intelligence algorithms for independent assessment of screening mammograms. JAMA Oncol. 2020;6:1581-1588. 32852536 10.1001/jamaoncol.2020.3321 PMC7453345

[37] Vela D, Sharp A, Zhang R, Nguyen T, Hoang A, Pianykh OS. Temporal quality degradation in AI models. Sci Rep. 2022;12:11654. 35803963 10.1038/s41598-022-15245-z PMC9270447

