# Supplementary material for: Artificial intelligence and consistency in patient care: a large-scale longitudinal study of mammographic density assessment
Source: BJR Artif Intell. 2025 Mar 3;2(1):ubaf004. doi: 10.1093/bjrai/ubaf004 (PMC11974406; doi:10.1093/bjrai/ubaf004)
Supplement: ubaf004_Supplementary_Data [file ubaf004_supplementary_data.docx]

**Supplemental Material**

***A. Approximation of BMI using data of image-based breast fat and thickness***

To address the lack of universal access to BMI collected during attendance at screening, we approximated BMI using image-based breast fat and thickness data, previously shown to provide a suitable alternative to clinically acquired weight and BMI [1]. To establish the relationship between BMI and image-based breast fat and thickness, we leveraged a subset of our study dataset consisting of 733 women that had weight and height data available which we used to calculate BMI. Image-based breast fat was computed for the screening examination of each woman using the area of non-dense tissue (non-dense area) calculated from mammographic views by the publicly-available LIBRA software [2,3]. Image-based breast thickness was extracted from the DICOM header of mammographic views. For each woman, image-based breast fat and thickness were averaged over all mammographic views of her screening examination to estimate per-woman breast fat and thickness values (Fig. S1). A linear regression analysis was then carried out to determine the relationship between BMI and the independent variables non-dense breast area and breast thickness (Table S1 and Fig. S2). Variable transformations were also explored to improve model fitness.

| 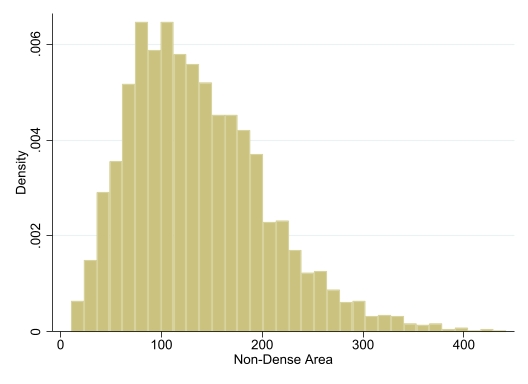 | 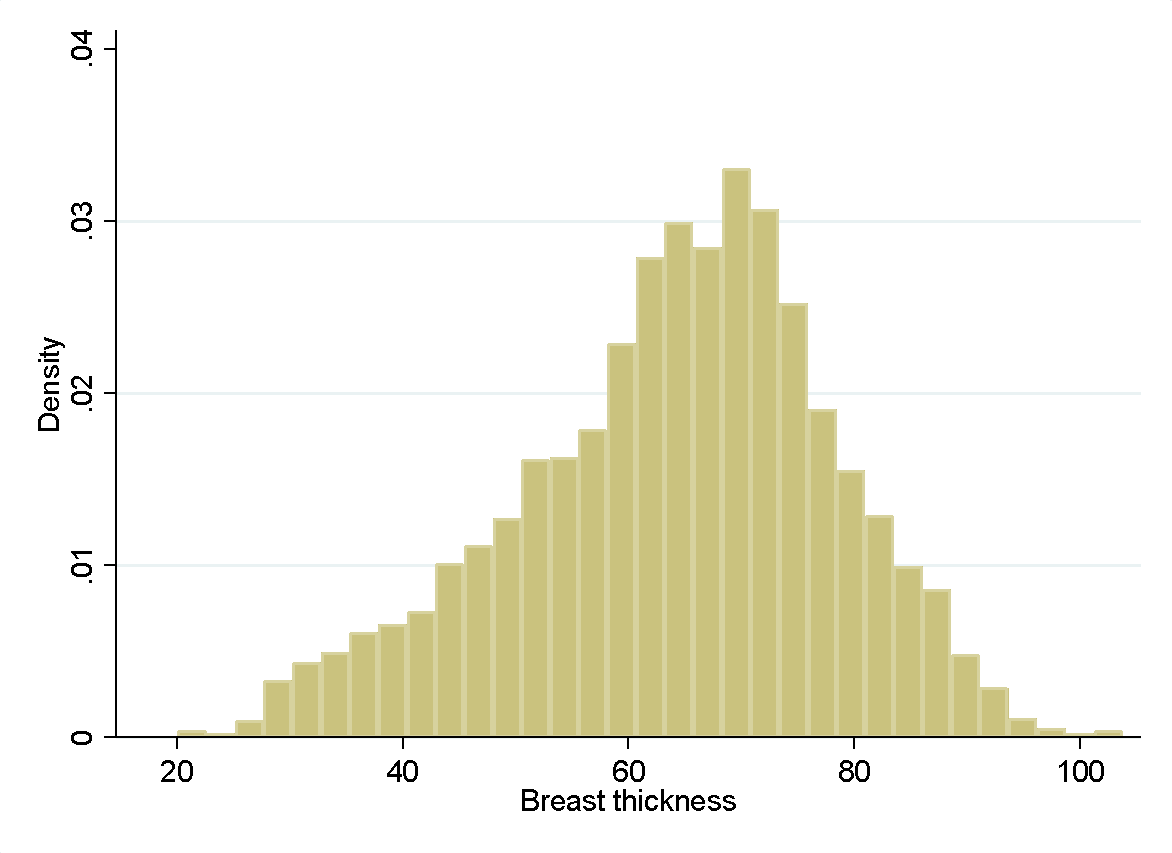 |
| --- | --- |
| 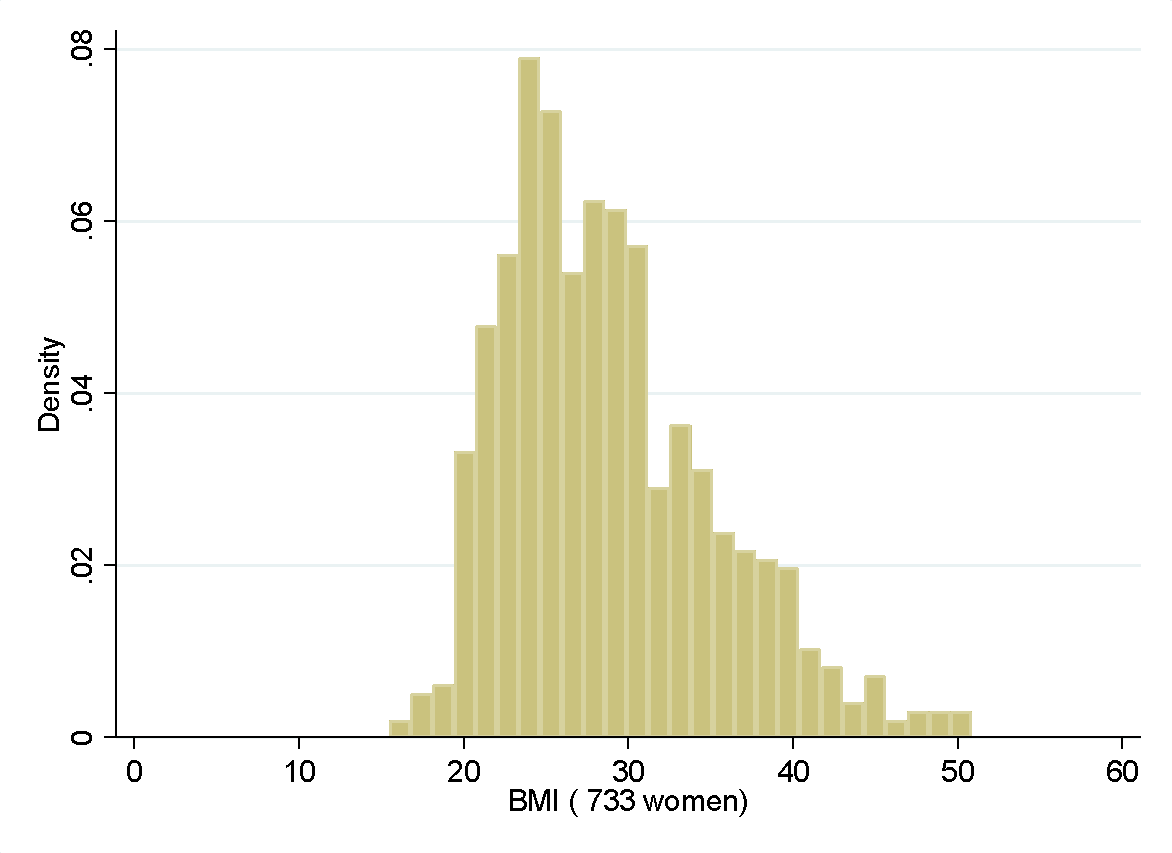 | |
| **Figure S1**. Distribution of BMI, non-dense area and breast thickness among 733 women included in BMI modeling. | |

**Table S1.** BMI model based on image-based non-dense area and breast thickness (R^2^ = 0.64; residual = 12.22).

|  | Coefficient | Std. err | p-value | 95% CI |
| --- | --- | --- | --- | --- |
| Non-dense area (sqrt) | 0.0394 | .00236 | <0.001 | 0.0348 – 0.0441 |
| Breast Thickness (sqrt) | 0.0779 | .00740 | <0.001 | 0.0634 – 0.0924 |
| Constant | 2.26 | 0.0449 | <0.001 | 2.17 – 2.34 |

| 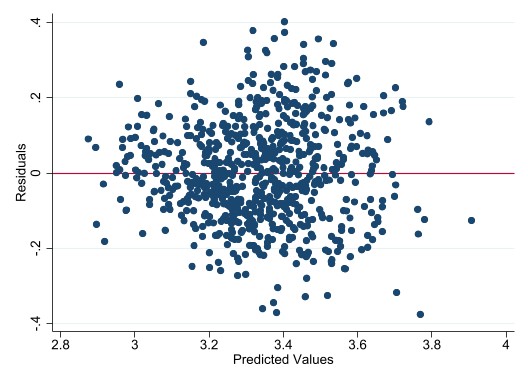 | 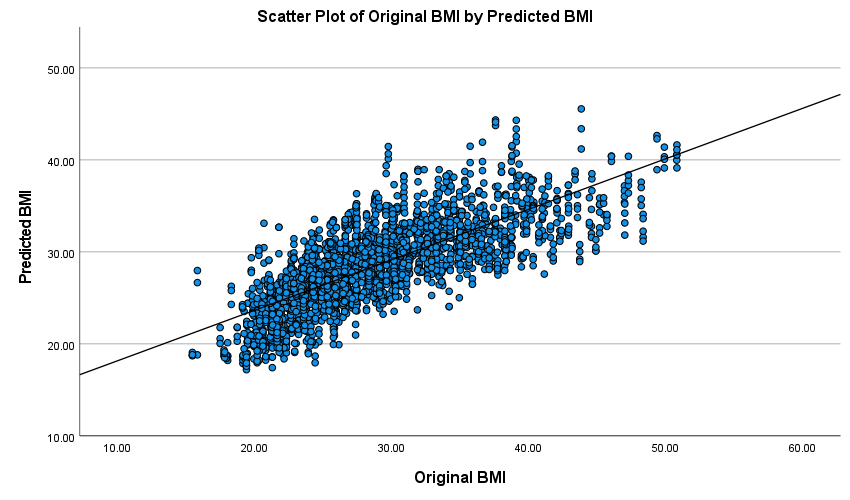 |
| --- | --- |
| **Figure S2**. (left) Residual plot showing differences between the original BMI values and the BMI values predicted from our linear regression model (right) among 733 women included in BMI modeling. | |

***B. Definition of subgroup of women with stable BMI***

From the established relationship between BMI and image-based breast fat and thickness, a predicted BMI value was derived for each woman of our study dataset using our linear regression model (Fig. S3). Subsequently, for each woman proportional BMI changes over time were estimated as $\frac{BMI_{\left\{ max \right\}}-BMI_{\left\{ min \right\}}}{BMI_{\left\{ min \right\}}}*100\%$. Women with stable BMI were then defined as women of proportional BMI changes <5% [4].

| 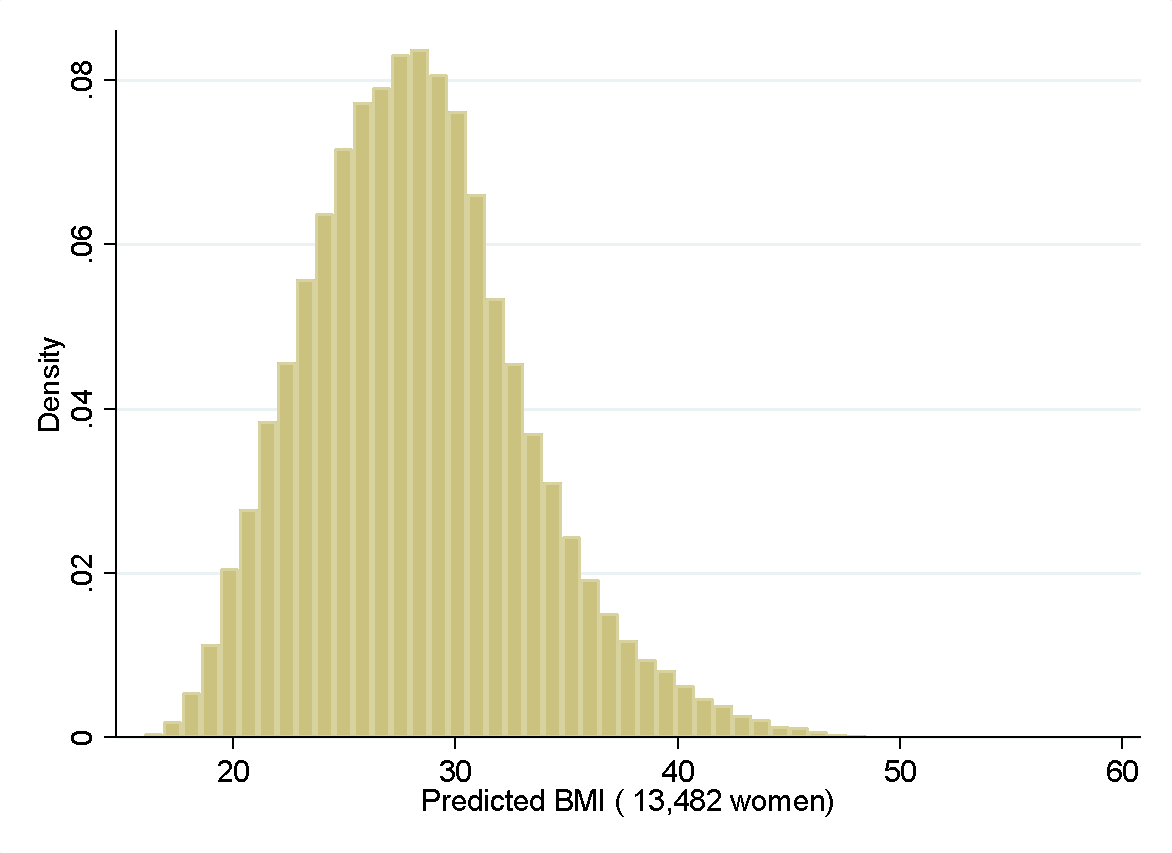 |
| --- |
| **Figure S3.** Histogram shows the distribution of predicted BMI results in study dataset. |

**References**

1. Nair, K.P.; Harkness, E.F.; Gadde, S.; Lim, Y.Y.; Maxwell, A.J.; Moschidis, E.; Foden, P.; Cuzick, J.; Brentnall, A.; Evans, D.G. The impact of using weight estimated from mammographic images vs. self-reported weight on breast cancer risk calculation. In Proceedings of the Medical Imaging 2017: Computer-Aided Diagnosis, 2017; pp. 740-745.

2. Keller, B.M.; Chen, J.; Daye, D.; Conant, E.F.; Kontos, D. Preliminary evaluation of the publicly available Laboratory for Breast Radiodensity Assessment (LIBRA) software tool: comparison of fully automated area and volumetric density measures in a case–control study with digital mammography. *Breast Cancer Research* **2015**, *17*, 1-17.

3. Gastounioti, A.; Kasi, C.D.; Scott, C.G.; Brandt, K.R.; Jensen, M.R.; Hruska, C.B.; Wu, F.F.; Norman, A.D.; Conant, E.F.; Winham, S.J. Evaluation of LIBRA Software for Fully Automated Mammographic Density Assessment in Breast Cancer Risk Prediction. *Radiology* **2020**, 192509.

4. Svärd, A.; Lahti, J.; Roos, E.; Rahkonen, O.; Lahelma, E.; Lallukka, T.; Mänty, M. Obesity, change of body mass index and subsequent physical and mental health functioning: a 12-year follow-up study among ageing employees. *BMC public health* **2017**, *17*, 1-10.
